# National series of long-term intensive harvesting trials in *Pinus radiata* stands in New Zealand: Initial biomass, carbon and nutrient pool data

**DOI:** 10.1016/j.dib.2019.104757

**Published:** 2019-11-06

**Authors:** L.G. Garrett, P.N. Beets, P.W. Clinton, S.J. Smaill

**Affiliations:** a Scion, Private Bag 3020, Rotorua, 3046, New Zealand; b Scion, PO Box 29237, Riccarton, Christchurch, 8440, New Zealand

**Keywords:** Sustainability, Nutrient, Harvest residue management, Organic matter removal, *Pinus radiata*, Planted forest

## Abstract

Global interest in addressing knowledge gaps relating to the effect of forest harvest intensity on soil fertility and long-term site productivity has resulted in the installation of numerous experiments, including Long-Term Site Productivity (LTSP) trials. To explore this issue in the context of the New Zealand planted forest estate, six LTSP sites were established from 1985 to 1994 across differing climate and soil conditions, then subjected to varying levels of organic matter removal during the harvest of the trees. Here we present data describing live above ground, forest floor and mineral soil carbon and nutrient pools immediately prior to, and following, harvesting at each site. Harvest residue management practices employed included the removal of stem only, whole tree, whole tree plus forest floor, whole tree plus forest floor and topsoil, and the addition of double harvest slash material. The data provides an understanding of biomass, carbon and nutrient pools at harvest and the impact of different harvest removal treatments on these pools. With the maturation of the trees at the LTSP sites, the data acquires even greater future value by enabling changes in soil properties to be quantified and correlated to variations in the biological properties at the site, including site productivity and critical microbial parameters. Overall, these data sets comprise a foundation for New Zealand to address the question – can the productivity of intensively managed planted *Pinus radiata* be maintained or enhanced through the judicious management of organic matter and nutrient pools over successive growing and harvesting cycles?

**Table:** Specifications Table

Subject area	Forestry
More specific subject area	Sustainable planted forest nutrient management
Type of data	Tables
How data was acquired	Field data collection, measurement and laboratory analysis. The biomass of whole live trees (excluding below ground), forest floor and sampling of the mineral soil.
Data format	Raw and analysed
Experimental factors	Harvest residue treatments include whole-tree harvest plus forest floor removed (FF), whole-tree harvest (WT), and stem only harvest (SO). Plus at selected sites the additional double slash (DS) and whole tree harvest plus forest floor and 2.5 cm topsoil removal (SR).
Experimental features	The data were collected from six Long-Term Site Productivity (LTSP) trials in New Zealand.
Data source location	New Zealand (latitude and longitude for each six LTSP trials in data tables)
Data accessibility	Analysed data sets are directly provided with this article.
Related research article	The most relevant articles follow: [[1], [2], [3]]

**Table:** 

**Value of the Data** • The data provides understanding of biomass, carbon and nutrient pools and the impact of harvest residue removal on planted forest productivity. • The data can be used to understand the future trajectory of the productive potential of New Zealand planted forests. • The data will serve as a foundation for future studies relating the resilience of soil properties to microbial community structure and function in forest soils. • The data adds to the global network of LTSP studies assessing the effects of forest harvest intensity on soil properties.

## 1 Data

**Fig. 1:**
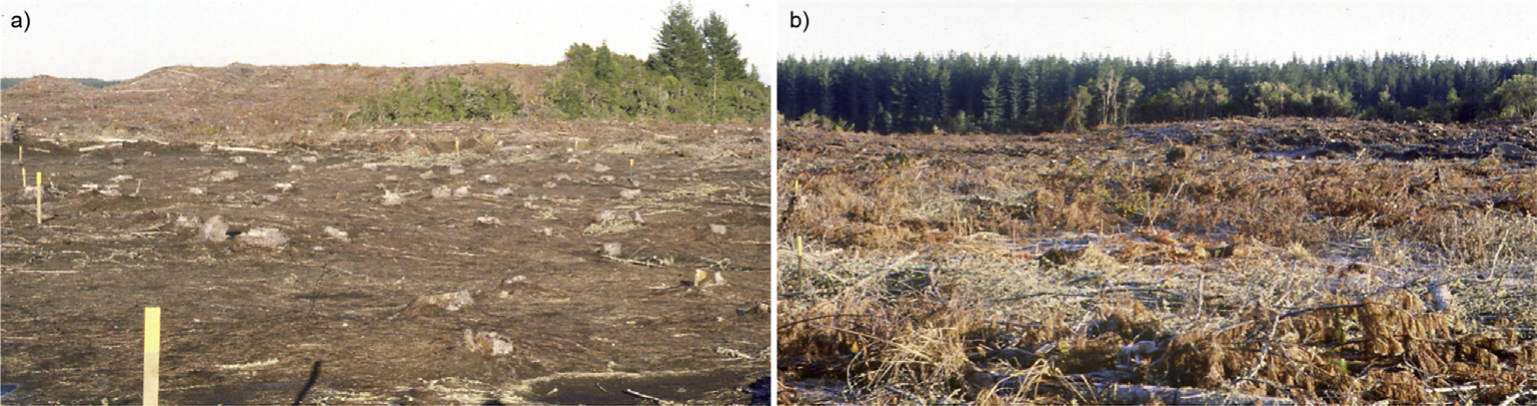
Image of two treatments showing the harvest residue left on site, a) FF treatment and b) SO treatment.

The dataset contains raw and analysed data collected from six Long-Term Site Productivity (LTSP) trials in New Zealand *Pinus radiata* forests, which are part of a global network of trials addressing knowledge gaps relating to the effect of forest harvest intensity on soil fertility and long-term site productivity [4]. Table 1 gives for each of the six New Zealand trials a description of the site and trial design and Fig. 1 shows an image of two harvest residue removal treatments.

**Table 1:** Site description and trial design (adapted from Smith et al. [5]).

Variable	Woodhill	Tarawera	Kinleith	Golden Downs	Burnham	Berwick
Trial ID	AK1029	FR44	FR188	FR220	FR128	FR127
Latitude	36°43′S	38°13′S	38°14′S	41°36′S	43°37′S	46°00′S
Longitude	174°24′E	176°00′E	175°58′E	172°53′E	172°19′E	170°01′E
Elevation (m asl)	100	90	490	450	70	200
MAT (°C)	14.3	14.0	13.2	10.4	11.5	10.3
Annual rainfall (mm)	1330	1820	1420	1340	639	747
Slope (degrees)	3	0	4	30	0	0
Soil parent material	Aeolian sand	Basaltic tephra	Pomaceous tephra	Moutere gravels	Pleistocene gravels	Loess derived from schist
Texture	sandy	gravelly, sandy loam, sand	sandy loam to silt loam	gravelly, silty clay loam over clay loam and clay	gravelly silt loam	silt loam
NZSC^a^ (USDA soil taxonomy^b^)	Typic Sandy Recent Soil (Psamment)	Tephric Recent Soil (Orthent)	Immature Orthic Pumice Soil (Vitrand)	Acidic Orthic Brown Soil (Dystrochrept)	Pallic Orthic Brown Soil (Ustochrept)	Mottled Fragic Pallic Soil (Ustochrept)
Planted (year)	1986	1989	1992^c^	1994	1990	1990
Initial tree spacing (m)	2 × 2	2 × 2	2 × 2	2 × 2	2 × 4	2 × 4
Organic matter removal treatments	SO, WT, FF, DS	SO, WT, FF, SR	SO, WT, FF	SO, WT, FF	SO, WT	SO, WT
Future other treatments	with and without fertiliser	with and without fertiliser	with and without fertiliser	with and without fertiliser	with and without fertiliser and weed control	with and without fertiliser and weed control
Split-plot size (m)	30 × 30	30 × 30	40 × 40	40 × 40	30 × 30	30 × 30
Number of replicate blocks	3	4	4	4	4	4
Number of main-plots	12	16	12	12	16	16
Number of split-plots	24	32	24	24	32	32
Lane width (m) between rows of plots	10	15	10	10	15	10 & 15

a NZSC (New Zealand Soil Classification) [6].

b Soil Taxonomy [7].

c The trial site was initially planted in 1991 but, due to a high mortality rate, was completely replanted in September 1992.

This data article contains two excel files:

1. Data file ‘NZ LTSP Pre-harvest’ contains six tables each on a separate excel sheet. Tables 2–5 present raw data (no standard error presented) and site average values (with standard error) for pre-harvest crop metrics, and ecosystem biomass, carbon and nutrient pools for the live tree, understory, forest floor and mineral soil. Tables 6, 7 presents raw data that was used to calculate site average values for understory, forest floor and mineral soil.
2. Data file ‘NZ LTSP Time zero’ contains six tables each on a separate excel sheet. Tables 8–13 present calculated data for each of the six sites for the biomass removed and remaining after different harvest residues removal treatments for biomass, carbon and nutrient pools.

## 2 Experimental design, materials, and methods

### 2.1 Intensive harvesting sites

The six New Zealand sites (Fig. 1) where the intensive harvesting trials were installed are described in Table 1. The trials were established as second rotation planted *P. radiata* stands, except Burnham which was third rotation planted forest. The previous rotation was also *P. radiata* with the exception of the Golden Downs site which was previously planted in *P. nigra*. The records for the first rotation planted forest at Burnham are not available. The land use before forests were planted was for Woodhill mobile sand dunes which was stabilised with marram grass prior to planting and Tarawera and Kinleith native cutover forest and shrubland. Both Golden Downs and Berwick were pastoral grazing which would have been produced from burning the native forest most likely around the turn of the century. It is uncertain of the land use prior to planting for Burnham.

To reduce within site variability as much as possible, stands growing on areas with uniform slope and aspect were selected for study. The previous rotation stands ranged in age from 26 to 44 years when harvesting occurred. This study was designed to test the hypotheses that 1) harvesting intensity is negatively correlated with second-rotation growth, 2) inadequate nutrition is the reason for the negative correlation, and 3) fertiliser additions can compensate for the negative impact of harvesting on growth. To avoid confounding organic matter removal treatments with other harvested related effects, such as soil compaction and differential weed growth among treatments, harvesting equipment was confined to designated access lanes between plots and complete weed control was undertaken. All sites also included treatments, future treatments, with or without the application of fertilisers, and the dryland sites included treatments with or without weed control.

### 2.2 Organic matter removal treatments

A split-plot randomised block design was used at each site with different organic matter removal treatments as main-plots that were split into sub-plots for future fertiliser addition (F) and no fertiliser addition (NF) treatments. There were four replications installed per site, except at Woodhill where only 3 replications were used. The organic matter removal treatments listed below were selectively applied at the six sites. The first two treatments were applied at all six sites, while the whole-tree harvesting plus forest floor removal treatments was applied at 4 sites. The remaining treatments were applied on a site-specific basis as indicated in Table 1:

• **Stem only harvesting (SO)** – conventional approach to forest harvesting which involved the removal of the merchantable stems off-site. Harvest residues (slash) including non-merchantable stems, branches, foliage, and cones were retained, along with forest floor, roots/stumps, and understorey vegetation from the previous stand.
• **Whole-tree harvesting (WT)** – bioenergy approach to forest harvesting which involved the removal of all stem material, and slash from crown material and understorey vegetation. Only the forest floor and roots/stumps from the previous stand were retained.
• **Whole-tree harvesting plus forest floor removal (FF)** – the removal of the merchantable stems and harvest residues, as in the WT treatment, and the removal of the forest floor, including fine roots growing within the forest floor. Only the roots/stumps growing in the soil were retained.
• **Double slash (DS)** – at Woodhill only. Involved stem only harvesting (as in the SO treatment), plus retention of double the amount of slash normally present after SO harvesting. The forest floor and roots/stumps from the previous stand were retained.
• **Whole tree harvest plus forest floor and 2.5 cm topsoil removal (SR)** – At Tarawera only. Involved the removal of merchantable stems, harvest residues, and forest floor from the previous stand followed by the removal of topsoil. Roots in topsoil were removed, while other roots/stumps from the previous stand were retained.

At some sites additional treatments were established and only monitored for tree growth, they were at Woodhill a management (standard forest company management of the day with no weed control) treatment with no windrowing and a management with windrowing, at Tarawera a management treatment, and at Kinleith a management (SO removal with no weed control) treatment and a management treatment with V-blading. At Berwick the treatment without weed control was later abandoned due to non-randomised distribution. The weed control treatment main-plots were randomly allocated. One weed control split-plot within the trial was installed outside its replicate block and so was not adjacent to the other split pair (reasons unknown).

### 2.3 Pre-harvest carbon and nutrient pools – end of first rotation

The biomass, carbon and nutrient pools of the tree crop, understory vegetation, forest floor, and mineral soil immediately prior to harvesting were directly measured at Woodhill (reported in Dyck et al. [1]), Tarawera, Kinleith, and Golden Downs trials. Tree crop biomass at Burnham and Berwick trials was not directly measured and were therefore estimated from stand data, using equations described in Madgwick [8], although other pools (forest floor, soil and understory) were directly measured. The following describes the method used to collect samples from the field. Woodhill live tree biomass methods are described in Dyck et al. [1].

#### 2.3.1 Biomass

The above-ground tree biomass (stem wood, stem bark, live and dead branch plus foliage, and cones) was determined using standard biomass procedures developed previously for *Pinus radiata* [9], with some modifications of the crown biomass procedure undertaken, as outlined below for each site. At Tarawera and Kinleith, 10 trees in a stand adjacent to the trial were selected for destructive sampling and at Golden Downs 30 trees were selected within 15 end-of-rotation tree measurement plots near the trial area and felled to determine individual component biomass and nutrient concentration. Trees were sampled by components in the live crown (1 year old foliage, 2 year old foliage and older foliage, live branches, dead branches, cones), dead portion of the crown (dead crown branches dead cluster and cones dead cluster cones) and stem (stem wood and stembark). Trees were felled and measured for total height and stem diameter over bark, at a base height of 0.15 m, and then every 6 m up the stem for Tarawera and Kinleith and 0.15 m, then every 5 m up the stem for Golden Downs. The live crown was divided into three equal length zones for branch sampling purposes. Diameter at the base of the green crown (10 cm below the chosen branch whorl) and a count of the number of branch whorls within each zone was also made. A random branch whorl per zone was selected and the number of branches counted in this whorl and in the whorls immediately above and below. A randomly selected sample branch was removed from the random branch whorl and the crown components (needles, live and dead branch matter and cones) separated, oven-dried to constant weight in a forced ventilation oven, and dry weights recorded. The branch counts were used to scale the mean weight of the sample branches to estimate the total weight of the crown components. A similar sampling procedure was used to estimate the oven dry mass of dead branches below the live crown. A 2.5 cm thick stem disc was collected at the base, and thereafter a 5 cm disc was collected at every over bark diameter measurement point. Diameter over and under bark and disc thickness (at four to eight points around the disc perimeter) were measured for each disc.

#### 2.3.2 Forest floor and soil

Forest floor material (combined LFH horizon, fresh litter (L), and partly and well decomposed litter (FH), LFH, <10 cm diameter) were collected before harvest at Tarawera, Kinleith (only LFH needles reported by Jones et al. [2] branches <10 cm diameter were excluded), Burnham and Berwick from each main-plot, and at Golden Downs from each split-plot. The LFH sample was collected using a randomly positioned 0.25 m^2^ sampling square from each collection area (main-plot or split-plot) from 4 points at Tarawera, 5 points at Kinleith, Burnham and Berwick and 1 point at Golden Downs, and bulked by sampling area. At Tarawera one of the replicated blocks was moved (considered too close to the stream) and forest floor and mineral soil samples were collected from the new block main-plots. No coarse woody debris was present.

Dyck et al. [1] reports on Woodhill mineral soil collection methods down to 1 m depth. Here we report that soil was collected at 0–0.1 and 0.1–0.9 m depth and reported down to 0.9 m. Soil was sampled for chemistry using a core sampler from 20 random points within each section (10 points bulked per sub-sample) for the 0–0.1 m depth interval and from 1 point for the 0.1–0.9 m depth interval. Bulk density samples were collected from five random points per section using a 5.3 cm internal diameter steel ring from the 0–0.1 cm depth interval and six samples were collect for the 0.1–0.9 m depth interval from across the site.

At Tarawera, the mineral soil was sampled for chemistry and bulk density at 6 random points within each main-plot using a 5.3 cm internal diameter steel ring from three depth intervals (0–0.05, 0–0.1, and 0.1–0.2 m). Deeper soil samples were collected by soil horizon layer (three horizon layers) down to 1 m depth from 2 of the points. The depth of each horizon layer was recorded and used to proportion bulk density into depth increments below 0.2 m depth (depth increments of 0.2–0.3 and 0.3–1 m). Chemistry results for these horizon sample depths were calculated into the standard depths by weighting the chemistry percent by the bulk density.

Jones et al. [2] reports on Kinleith mineral soil collection down to 0.3 m. Here we report on the additional collection methods down to 1 m soil depth. Soil was sampled for chemistry at 10 random points within each main-plot using a Hoffer tube sampler from three depth intervals (0–0.1, 0.1–0.2, 0.2–0.3 m), bulked by main-plot and by block for selected analysis. Deeper soil samples were collected (0.3–0.5, and 0.5–1 m) from 4 of the points and bulked two across two blocks (bulked blocks 1 and 2, and block 3 and 4). Bulk density samples were collected from one random point per main-plot using a 5.3 cm internal diameter steel ring from three depth intervals (0–0.1, 0.1–0.2, 0.2–0.3 m) and from one point per main-plot from replicated block 1 and 3 only for deeper soil (0.3–0.5, and 0.5–1 m).

At Golden Downs, the mineral soil was very gravely and therefore sampled for chemistry and bulk density at 6 locations within the trial area using a sampling pit (342 × 198 mm) at two depths (0–0.3 and 0.3–0.6 m). Each sample was separated in the field into >6 mm rock fraction (weighed and discarded) and <6 mm fraction (weighed, sub-sampled and weighed).

At Burnham, the mineral soil was sampled for chemistry at 20 random points within each main-plot using a core sampler from two depth intervals (0–0.1, and 0.1–0.2 m), bulked by main-plot. Deeper soil samples were collected from four main-plots only (one main-plot per block) using a pit (0.2–0.3, and 0.3–0.5 m) from one of the points. Bulk density samples were collected from one random point per main-plot using a 5.3 cm internal diameter steel ring from four depth intervals (0–0.1, 0.1–0.2, 0.2–0.3, and 0.3–0.5 m).

At Berwick, the mineral soil was sampled for chemistry at 20 random points within each main-plot using a Hoffer tube sampler from two depth intervals (0–0.1, and 0.1–0.2 m), bulked by main-plot. Deeper soil samples were collected (0.2–0.4, and 0.4–0.6 m) from one of the points in each main-plot. Bulk density samples were collected from one random point per main-plot using a 5.3 cm internal diameter steel ring from four depth intervals (0–0.1, 0.1–0.2, 0.2–0.4, and 0.4–0.6 m).

#### 2.3.3 Understorey

There was very little understorey vegetation at the Tarawera and Golden Downs sites, therefore this component was ignored. The understorey at Kinleith was comprised of New Zealand indigenous tree hardwood species and tree ferns and was measured from a sub-set of ten randomly selected main-plots using one 4 × 3 m sampling square randomly located within each selected main-plot. The understorey at Burnham was comprised of eucalypts and acacia seedlings and Berwick was comprised of ferns, honeysuckle, gorse, coprosma, grass, and *P. radiata* seedlings. The understory from Burnham and Berwick were collected from all 16 main-plots (60 × 30 m) using three 2 × 2 m sampling squares randomly located within each main-plot. All above ground vegetation was collected and bulked by main-plot. The understorey at Woodhill was pampas grass and sampling is described in Dyck et al. [1].

### 2.4 Sample preparation and analysis

#### 2.4.1 Biomass and forest floor

Live biomass (stem wood, stem bark, live and dead branch plus foliage, and cones), forest floor (LFH) and understory samples were oven dried at 70 °C to constant weight and weighed. Foliage was then separated from branch samples, oven dried to constant weight and weighed. Stem, stem bark, and branch components, and understory were chipped then ground to pass a Wiley mill 2 mm screen mesh. Foliage was ground in a Wiley mill to pass a 1 mm screen. Forest floor was ground in a Wiley mill to pass a 2 mm screen. Biomass components for Tarawera were bulked across all trees to make one tree component per site. At Golden Downs branch plus cone and all foliage was bulked to a site level for each sampling zone. Biomass from other sites were not bulked. There was no chemical analysis of the Tarawera new block installation forest floor. Where samples had not been tested for total carbon and total nitrogen using a LECO FPS-21000 CNS thermal combustion furnace they were retrieved from the archive and tested. Other analysis includes phosphorous, calcium, potassium, magnesium, boron and copper by wet digestion [10]. For Woodhill forest floor only a range of elements (Ca, K, Mg, Mn, B, Zn) was determined by X-ray fluorescence (XRF). Forest floor samples were also tested for loss on ignition at 525 °C. Understory samples were not archived therefore understory samples from Woodhill, Kinleith, and Burnham have no total carbon analysis and total nitrogen was measured using wet digestion methods [10]. Berwick understory samples were historically tested for total carbon and total nitrogen using a LECO FPS-21000 CNS thermal combustion furnace.

#### 2.4.2 Soil

Mineral soil samples were air-dried (<40 °C) and then sieved to attain the <2 mm fraction for analysis and bulk density calculations. The soil bulk density sample was oven dried at 104 °C and weighed. Soil chemical samples were retrieved from the archive and tested for total carbon and total nitrogen using a LECO FPS-21000 CNS thermal combustion furnace for all sites except Burnham. Retrieved soil samples for Woodhill, Tarawera and Berwick were also tested for total phosphorus using flow injection analysis (FIA) colorimetry after sulphuric acid digest. For all sites and some soil depths total phosphorus, inorganic and organic phosphorus, calcium, potassium, magnesium, sodium and a range of other elements (Zn, Cu, Fe, Mn, S, Al, and B) were measured by XRF spectrometry. Woodhill XRF analysis was undertaken pre-1990. Post 1990 all remaining XRF analysis was done plus additional Woodhill XRF analysis to capture total phosphorus, inorganic and organic phosphorus and zinc. Following methods described in Nicholson [10] most samples were also tested for pH using an electronic probe, 1:2.5 w/v, exchangeable cations (calcium, potassium, magnesium and sodium) measured by flame atomic absorption spectrophotometry (AAS) after leaching with a 1 N ammonium acetate solution followed by the addition of strontium solution, cation exchange capacity (CEC) measured on an autoanalyzer by colorimetry after leaching with a 1 N ammonium acetate solution followed by a 1 N sodium chloride solution, available phosphorus measured on an autoanalyzer by colorimetry after sequential Bray 2 reagent (NH_4_F/HCl) extraction.

### 2.5 Pre-harvest data and statistical analysis

Pre-harvest ecosystem biomass, carbon and nutrient pools are reported for each site as a mean and standard error for live above ground (foliage, branch, stem bark and stem wood), forest floor (LFH) and soil (0–0.1 m, where possible 0.1–0.3, 0–0.3 m, and to the deepest measurement depth).

#### 2.5.1 Above ground tree biomass

The total above-ground live tree biomass per site on a per hectare basis was estimated using the basal area ratio method [11] which were applied to the measured tree basal area at each trial site. For Tarawera and Kinleith this method was further refined for the tree crown by using the ratio of the cross-sectional areas of the stems at the base of the green crown and at DBH to allow for biomass sample trees to have come from an area adjacent to the experimental site. This method used the treatment area tree measurements (Woodhill used plots from the site area but did not exactly correspond to the treatment area) and measured live tree biomass data to generate per hectare site area-based biomass dry matter, carbon and nutrient amounts. At Burnham and Berwick the biomass dry-matter was not directly measured and were therefore estimated from stand data from the site area, using generalised equations described in Madgwick [8] with crown mass calculated as described for Tarawera and Kinleith. The generalised equations produced stem (wood plus bark), live foliage and live branch mass estimates. The mass of bark at both Burnham and Berwick was determined using a stem bark to stem wood mass ratio of 0.12 which came from measured data at the end of second rotation LTSP Berwick biomass (no fertiliser addition treatment) (unpublished data). For Burnham and Berwick total carbon and total nitrogen concentrations (using a LECO FPS-21000 CNS thermal combustion furnace) by tree component used the mean end of second rotation LTSP Berwick biomass (no fertiliser addition treatment) (unpublished data). Phosphorus, potassium, calcium, magnesium and boron concentrations were measured for foliage, branch and stem (wood + bark) using methods described in section 2.4 sample preparation and analysis from limited samples collected from each site, Burnham and Berwick, post-harvest. All pools are reported on a slope corrected basis. Standard errors are not reported because the biomass data were obtained from an adjacent stand near by the trial site. Above ground live tree biomass for Burnham and Berwick was estimated using the biomass equations from Madgwick [11] which were applied to the measured tree basal area at each trial site. All above ground tree biomass is reported by tree component (live foliage, live branches, dead branches, cones, stem bark and stem wood, plus below ground roots >2 mm for Woodhill). The stem wood and stem bark pools are reported to ground level. The above-ground live tree component concentrations are reported as weight adjusted concentrations, i.e. amount of carbon or nutrient on a per hectare basis divided by the by the mass per hectare of each tree component.

#### 2.5.2 Forest floor and soil

Forest floor mass pools were calculated using total oven-dry ash free mass, carbon and nutrient stocks by the respective concentration and the total oven-dry mass divided by the total collection area. Forest floor concentrations are reported oven-dry ash free. Mineral soil pools were calculated for the fine earth fraction (<2 mm) by sampling depth range by multiplying the concentration data with the corresponding density data. All pools are reported by area on a slope corrected basis. Soil carbon and nutrient concentrations are reported as measured.

### 2.6 Harvesting – method of organic matter removal

Harvesting of the trial series was undertaken with care to reduce compaction and disturbance within the treatment plot areas. This was done by designing access lanes between rows of plots to keep machine traffic off plots and allow for felled trees to be winched out of the plots. The slash in the SO and DD (Woodhill only) were reduced in size using a chainsaw and more evenly distributed over the plot. Forest floor was removed pre-harvest either by hand using garden rakes (Kinleith, Golden Downs) or with the use of a farm tractor with a rear mounted blade (Tarawera) or a combination of both (Woodhill; inner 5 m strip hand removed). Post-harvest any debris was removed from the plots by hand.

### 2.7 Post-harvest carbon and nutrient pools

Biomass, carbon and nutrient pools retained on site following application of the various harvest/site preparation treatments were calculated from the pre-harvest data to generate ecosystem pools for each harvest residue retention treatment. The post-harvest pools are reported as removed biomass (stem, tree crown, understory, forest floor), harvest residue biomass (remaining live above ground tree, understory, forest floor), mineral soil (0–10, 10–30, 30–100 cm; or to deepest sampling depth), and for Woodhill only below ground roots >2 mm.

The percent of the stem (wood plus bark) left on site as the above ground stump was set for all sites at 2.1% of the stem, which is based on actual measurements at the Woodhill site. There is an assumption that 2.1% of the stem remained on site as the stump at the other sites. An assumption on the level of harvest residues removed were made as not 100% of all material could possibly be removed. It was assumed that with WT harvest 5% of tree foliage would remain on site, with FF harvest it was assumed that 10% of the forest floor would remain on site, with DS harvest it was assumed that double the amount of SO treatment stem, foliage, branches and cones were added to the site. For SO treatment is was assumed that 10% of the stem (wood and bark) minus the stump fraction (2.1%, based on measurement at Woodhill) would remain on site (equalling 7.9% stem and bark). For sites where weed biomass was present and measured it was assumed that 20% of the understory biomass was left on site after harvest for FF and WT treatments, 100% for SO treatment and 150% for DS treatment. All above and below ground biomass removed and harvest residues are reported at the site level.

Where soil was collected by main-plot, the treatment average carbon and nutrient pool values are reported for each treatment. Where soil was collected by site, the site average carbon and nutrient pool values are used across treatments. Tarawera SR treatment pools for soil were not assessed and therefore the site average is used to generate pool estimates. The carbon and nutrient pools for the Tarawera SR treatment used 50% of the 0–0.05 m soil pool.
